# Supplementary material for: The Encapsulation of Citicoline within Solid Lipid Nanoparticles Enhances Its Capability to Counteract the 6-Hydroxydopamine-Induced Cytotoxicity in Human Neuroblastoma SH-SY5Y Cells
Source: Pharmaceutics. 2022 Aug 30;14(9):1827. doi: 10.3390/pharmaceutics14091827 (PMC9506317; doi:10.3390/pharmaceutics14091827)

SUPPLEMENTARY MATERIALS

FOR

Encapsulation of Citicoline within Solid Lipid Nanoparticles enhances its capability to counteract the 6-hydroxydopamine-induced cytotoxicity in human neuroblastoma SH-SY5Y cells

BY

Andrea Margari[1,2], Anna Grazia Monteduro[1,2], Silvia Rizzato[1,2], Loredana Capobianco[3], Alessio Crestini[4], Roberto Rivabene[4], Paola Piscopo[4], Mara D'Onofrio[5], Valeria Manzini[5], Giuseppe Trapani[6], Alessandra Quarta[2], Giuseppe Maruccio[1,2], Carmelo Ventra [7], Luigi Lieto [7], and Adriana Trapani[6]

[1] Omnics Research Group, Department of Mathematics and Physics "Ennio De Giorgi", University of Salento and INFN Sezione di Lecce, Via per Monteroni, 73100, Lecce, Italy

[2]CNR-NANOTEC Institute of Nanotechnology, Via Monteroni, 73100 Lecce, Italy

[3] Department of Biological and Environmental Sciences and Technologies, University of Salento, 73100 Lecce, Italy

[4] Department of Neuroscience-Istituto Superiore di Sanità-viale Regina Elena 299-00161-Rome, Italy

[5] European Brain Research Institute (EBRI) "Rita Levi-Montalcini", Rome, Italy; viale Regina Elena 295-00161-Rome, Italy

[6]Department of Pharmacy-Drug Sciences, University of Bari "Aldo Moro", via Orabona, 4-70125 Bari, Italy; adriana.trapani@uniba.it (A.T.)

[7] Esseti Farmaceutici, Via Cavalli di Bronzo, 39-46, 80046 San Giorgio a Cremano, Naples, Italy

Excel S1: RESULTS ANALYSIS OF THE TEM SIZE OF MORE THAN 100 PARTICLES BY IMAGE J SOFTWARE

	Label	Length
1	Particle 1	869.300
2	Particle 2	980.235
3	Particle 3	757.701
4	Particle 4	775.779
5	Particle 5	813.907
6	Particle 6	448.884
7	Particle 7	155.482
8	Particle 8	280.505
9	Particle 9	272.350
10	Particle 10	355.064
11	Particle 11	541.527
12	Particle 12	244.346
13	Particle 13	231.271
14	Particle 14	231.621
15	Particle 15	389.596
16	Particle 16	304.379
17	Particle 17	855.767
18	Particle 18	497.907
19	Particle 19	295.291
20	Particle 20	257.517

21	Particle 21	209.383
22	Particle 22	309.063
23	Particle 23	407.352
24	Particle 24	185.432
25	Particle 25	227.424
26	Particle 26	294.065
27	Particle 27	313.737
28	Particle 28	347.375
29	Particle 29	506.896
30	Particle 30	363.849
31	Particle 31	224.129
32	Particle 32	214.291
33	Particle 33	422.589
34	Particle 34	408.508
35	Particle 35	120.728
36	Particle 36	141.858
37	Particle 37	102.488
38	Particle 38	94.409
39	Particle 39	321.994
40	Particle 40	91.454
41	Particle 41	87.756
42	Particle 42	187.963
43	Particle 43	278.597
44	Particle 44	102.549
45	Particle 45	135.019
46	Particle 46	81.024
47	Particle 47	82.299
48	Particle 48	92.005
49	Particle 49	108.501
50	Particle 50	224.773
51	Particle 51	214.817

52	Particle 52	324.301
53	Particle 53	114.989
54	Particle 54	100.837
55	Particle 55	236.777
56	Particle 56	144.767
57	Particle 57	152.767
58	Particle 58	195.312
59	Particle 59	395.284
60	Particle 60	296.506
61	Particle 61	254.019
62	Particle 62	208.254
63	Particle 63	289.123
64	Particle 64	99.848
65	Particle 65	93.205
66	Particle 66	238.224
67	Particle 67	184.126
68	Particle 68	148.064
69	Particle 69	68.993
70	Particle 70	132.362
71	Particle 71	169.155
72	Particle 72	377.489
73	Particle 73	205.859
74	Particle 74	102.052
75	Particle 75	630.662
76	Particle 76	476.518
77	Particle 77	105.302
78	Particle 78	241.550
79	Particle 79	84.735
80	Particle 80	91.580
81	Particle 81	110.275
82	Particle 82	108.568

83	Particle 83	103.660
84	Particle 84	130.620
85	Particle 85	127.295
86	Particle 86	142.896
87	Particle 87	241.106
88	Particle 88	295.541
89	Particle 89	254.245
90	Particle 90	308.342
91	Particle 91	332.531
92	Particle 92	250.473
93	Particle 93	341.119
94	Particle 94	406.560
95	Particle 95	245.190
96	Particle 96	307.897
97	Particle 97	246.850
98	Particle 98	232.027
99	Particle 99	314.227
100	Particle 100	228.042
101	Particle 101	120.767
102	Particle 102	117.932
103	Particle 103	128.377
104	Particle 104	121.562
105	Particle 105	87.087
106	Particle 106	85.770
107	Particle 107	104.238

108	Particle 108	87.120
109	Particle 109	84.122
110	Particle 110	377.066
111	Particle 111	189.108
112	Particle 112	780.238
113	Particle 113	427.928
114	Particle 114	818.428
115	Particle 115	807.828
116	Particle 116	511.949
117	Particle 117	366.276
118	Particle 118	244.255
119	Particle 119	134.592
120	Particle 120	272.707
	Mean	276.819
	SD	198.225
	Min	68.993
	Max	980.235

Excel S2: RESULTS OBTAINED FROM THE STATISTICAL APPROACH DESCRIBED IN SECTION 2.8

	CTRL NT	6OHDA 0.04 mM	0.05 mM Cit	0.05 mM SLN-Cit	0.05 mM Plain-SLNs	0.06 mM Cit	0.06 mM SLN-Cit
Exp 1 replicates	0,750	0,618	0,640	0,721	0,622	0,670	0,677
Exp 1 replicates	0,718	0,652	0,615	0,760	0,618	0,622	0,701
Exp 1 replicates	0,701	0,719	0,691	0,724	0,711	0,652	0,692
Exp 2 replicates	0,805	0,700	0,700	0,770	0,738	0,618	0,749
Exp 2 replicates	0,848	0,626	0,701	0,806	0,699	0,761	0,750
Exp 2 replicates	0,805	0,59	0,652	0,705	0,647	0,7	0,732
Exp 3 replicates	0,482	0,406	0,425	0,523	0,415	0,393	0,486
Exp 3 replicates	0,546	0,416	0,415	0,556	0,506	0,453	0,508
Exp 3 replicates	0,531	0,36	0,387	0,5	0,428	0,367	0,474
Exp 4 replicates	0,584	0,554	0,578	0,575	0,582	0,514	0,588
Exp 4 replicates	0,59	0,547	0,566	0,6	0,57	0,605	0,572
Exp 4 replicates	0,565	0,57	0,623	0,605	0,562	0,573	0,538
Exp 5 replicates	0,378	0,31	0,298	0,319	0,319	0,315	0,322
Exp 5 replicates	0,396	0,357	0,34	0,376	0,332	0,33	0,376
Exp 5 replicates	0,379	0,325	0,326	0,353	0,334	0,306	0,325

	CTRL NT	6OHDA 0.04 mM	0.05 mM Cit	0.05 mM SLN-Cit	0.05 mM Plain-SLNs	0.06 mM Cit	0.06 mM SLN-Cit
Meane Exp 1	0,723	0,663	0,649	0,735	0,650	0,648	0,690
Meane Exp 2	0,819	0,639	0,684	0,760	0,695	0,693	0,744
Meane Exp 3	0,520	0,394	0,409	0,526	0,450	0,404	0,489
Meane Exp 4	0,580	0,557	0,589	0,593	0,571	0,564	0,566
Meane Exp 5	0,384	0,331	0,321	0,349	0,328	0,317	0,341

	CTRL NT	6OHDA 0.04 mM	0.05 mM Cit	0.05 mM SLN-Cit	0.05 mM Plain-SLNs	0.06 mM Cit	0.06 mM SLN-Cit
Number of values	5	5	5	5	5	5	5

Minimum	0,3843	0,3307	0,3213	0,3493	0,3283	0,317	0,341
25% Percentile	0,452	0,3623	0,3652	0,4378	0,389	0,3607	0,4152
Median	0,5797	0,557	0,589	0,5933	0,5713	0,564	0,566
75% Percentile	0,7712	0,6508	0,6665	0,7477	0,6725	0,6705	0,7168
Maximum	0,8193	0,663	0,6843	0,7603	0,6947	0,693	0,7437
Mean	0,6052	0,5167	0,5305	0,5929	0,5389	0,5253	0,566
Std. Deviation	0,1706	0,148	0,1578	0,1673	0,15	0,1602	0,1608
Std. Error	0,07632	0,06617	0,07056	0,07483	0,06709	0,07166	0,07192
Lower 95% CI	0,3933	0,333	0,3346	0,3851	0,3526	0,3263	0,3663
Upper 95% CI	0,8171	0,7004	0,7264	0,8006	0,7252	0,7242	0,7657

Table Analyzed	Data 6
Repeated Measures ANOVA	
P value	< 0,0001
P value summary	***
Are means signif. different? (P < 0.05)	Yes
Number of groups	7
F	8,996
R square	0,6922
Was the pairing significantly effective?	
R square	0,9297
F	258
P value	< 0,0001
P value summary	***
Is there significant matching? (P < 0.05)	Yes

ANOVA Table	SS	df	MS
Treatment (between columns)	0,03639	6	0,006064
Individual (between rows)	0,6956	4	0,1739
Residual (random)	0,01618	24	0,0006741
Total	0,7482	34	

Bonferroni's Multiple Comparison Test	Mean Diff,	t	Significant? P < 0,05?	Summary	95% CI of diff
CTRL NT vs 6OHDA 0.04 mM	0,08853	5,392	Yes	***	0,03277 to 0,1443
CTRL NT vs 0.05 mM Cit	0,07473	4,551	Yes	**	0,01897 to 0,1305
CTRL NT vs 0.05 mM SLN-Cit	0,01233	0,7511	No	ns	-0,04343 to 0,06810
CTRL NT vs 0.05 mM Plain-SLNs	0,06633	4,04	Yes	*	0,01057 to 0,1221
CTRL NT vs 0.06 mM Cit	0,07993	4,868	Yes	**	0,02417 to 0,1357
CTRL NT vs 0.06 mM SLN-Cit	0,0392	2,387	No	ns	-0,01656 to 0,09496
6OHDA 0.04 mM vs 0.05 mM Cit	-0,0138	0,8404	No	ns	-0,06956 to 0,04196
6OHDA 0.04 mM vs 0.05 mM SLN-Cit	-0,0762	4,641	Yes	**	-0,1320 to -0,02044
6OHDA 0.04 mM vs 0.05 mM Plain-SLNs	-0,0222	1,352	No	ns	-0,07796 to 0,03356
6OHDA 0.04 mM vs 0.06 mM Cit	-0,0086	0,5237	No	ns	-0,06436 to 0,04716
6OHDA 0.04 mM vs 0.06 mM SLN-Cit	-0,04933	3,004	No	ns	-0,1051 to 0,006430
0.05 mM Cit vs 0.05 mM SLN-Cit	-0,0624	3,8	Yes	*	-0,1182 to -0,006636
0.05 mM Cit vs 0.05 mM Plain-SLNs	-0,0084	0,5116	No	ns	-0,06416 to 0,04736
0.05 mM Cit vs 0.06 mM Cit	0,0052	0,3167	No	ns	-0,05056 to 0,06096
0.05 mM Cit vs 0.06 mM SLN-Cit	-0,03553	2,164	No	ns	-0,09130 to 0,02023
0.05 mM SLN-Cit vs 0.05 mM Plain-SLNs	0,054	3,289	No	ns	-0,001764 to 0,1098
0.05 mM SLN-Cit vs 0.06 mM Cit	0,0676	4,117	Yes	**	0,01184 to 0,1234
0.05 mM SLN-Cit vs 0.06 mM SLN-Cit	0,02687	1,636	No	ns	-0,02890 to 0,08263
0.05 mM Plain-SLNs vs 0.06 mM Cit	0,0136	0,8282	No	ns	-0,04216 to 0,06936
0.05 mM Plain-SLNs vs 0.06 mM SLN-Cit	-0,02713	1,652	No	ns	-0,08290 to 0,02863
0.06 mM Cit vs 0.06 mM SLN-Cit	-0,04073	2,481	No	ns	-0,09650 to 0,01503

FURTHER TEM IMAGES THAT CONTAIN MANY SMALL PARTICLES AND DENOTE THE BROAD DISTRIBUTION OF THE SLNs

Figure S1. Additional TEM images of CIT-SLNs

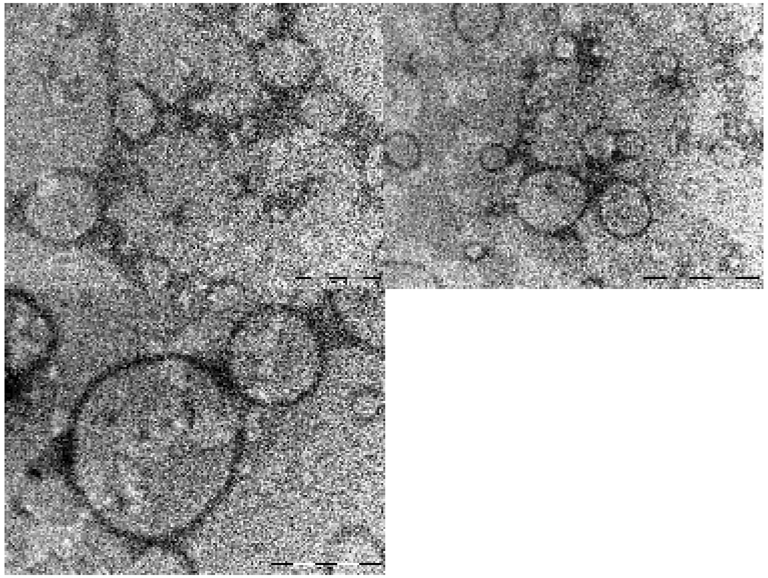

Supplement: Supplementary file 1 [file pharmaceutics-14-01827-s001.zip › pharmaceutics-1869670-supplementary.pdf]
